# Gene expression analyses on multi-target mode of action of black cohosh in menopausal complaints – a pilot study in rodents

**DOI:** 10.1007/s00404-021-06105-8

**Published:** 2021-07-14

**Authors:** Petra Stute, Stefan Ehrentraut, Hans-Heinrich Henneicke-von Zepelin, Petra Nicken

**Affiliations:** 1 Department of Obstetrics, Gynaecologic Endocrinology and Reproductive Medicine, University Women’s Hospital, University Clinic of Bern, Friedbuehlstrasse 19, 3010 Bern, Switzerland; 2 Arrows Biomedical Deutschland GmbH, Münster, Germany; 3 Department of Regulatory Affairs, Schaper and Brümmer GmbH and Co. KG, Salzgitter, Germany

**Keywords:** Cimicifuga racemosa, Black cohosh, Menopausal symptoms, Brain, Gene expression, Mode of action

## Abstract

**Purpose:**

This study aimed at assessing gene expression profiles in hippocampus and hypothalamus of ovariectomized (OVX) rats with or without treatment with an isopropanolic extract of *Cimicifuga racemosa* rhizomes (iCR) in comparison to intact rats.

**Methods:**

Exploration of hippocampal (Hi) and hypothalamic (Hy) tissue from Sprague Dawley rats: without OVX (N_Hi_ = N_Hy_ = 4), tissues 3 months after OVX (N_Hi_ = 4, N_Hy_ = 3), or tissues of rats after their treatment with iCR for 3 months after OVX (N_Hi_ = N_Hy_ = 2). Gene expression profiles in these tissues were investigated by RNA-microarray-analysis and subsequent verification by qPCR.

**Results:**

4812 genes were differentially regulated when comparing the three groups in hippocampus and hypothalamus. iCR compensated the effects of OVX in 518 genes. This compensatory effect was most prominent in hippocampal signalling pathways, thereof genes (GAL, CALCA, HCRT, AVPR1A, PNOC, etc.) involved in thermoregulation, regulation of sleep and arousal, blood pressure regulation, metabolism, nociception, hormonal regulation, homeostasis, learning and cognition, mood regulation, neuroendocrine modulation, etc.. In the hypothalamus, iCR compensated OVX-effects at TAC3 and OPRM1 but not at KISS1. These genes are involved in the pathophysiology of hot flashes.

**Conclusions:**

Our pilot study findings support a multifaceted mode of action of iCR in menopausal complaints on a tissue-specific brain gene expression level.

**Supplementary Information:**

The online version contains supplementary material available at 10.1007/s00404-021-06105-8.

## Introduction

Menopause-related oestrogen decline affects the aging process of the female brain and is accompanied by a progressive decline in cognition and memory, respectively [1]. Furthermore, oestrogen decline also leads to a dysfunction of temperature-sensitive neurons in the hypothalamic preoptic area and other hypothalamic nuclei areas. This may explain the reduced ability to adapt to changes in ambient temperature resulting in hot flushes, the most common menopausal symptom. The hippocampus plays a major role in learning and memory. However, it also contributes to attention, arousal, and emotional states including stress, and to adaptive and reproductive behaviour. Hippocampal outputs are predominantly inhibitory on downstream neuroendocrine activity [2].

During menopause, oestrogens or herbs such as black cohosh are frequently used to treat hot flushes. One aspect in the mode of action may be that oestrogens as well as black cohosh increase c-fos protein (marker of neuronal activity) positive cell density within the hypothalamic nuclei [3]. Furthermore, black cohosh also interacts with the hypothalamic–pituitary–adrenal axis and the sympathetic adrenomedullary system under stress conditions leading to alleviation of acute stress responses in rats [4]. Its neuroprotective effect was recently studied using Sprague Dawley rats comparing the impact of oestradiol and black cohosh on hypothalamic nuclei with ovariectomized (OVX) and intact rats. It was shown that both oestradiol and black cohosh restored the function or prevented dysfunction of the hypothalamic neurons, albeit through different mechanisms. Black cohosh exerted changes on EEG brain activity similar to those changes exerted by drugs used to treat Parkinson's disease (Selegiline), dementia (Tacrine), or depression (Moclobemide) and to influence mood and cognition (Ginkgo) [5].

In an exploratory pilot study using RNA-microarray-analysis and subsequent verification by qPCR, we assessed gene expression profiles in the hippocampus and hypothalamus of ovariectomized (OVX) rats with or without black cohosh treatment compared to intact rats. Our hypothesis was that black cohosh can, at least partly, compensate OVX-induced alterations of the hippocampal and hypothalamic gene expression profiles compared to intact rats.

## Material and methods

### Animal characteristics and treatment

Hippocampal and hypothalamic tissues from female Sprague Dawley rats (total *n* = 20), treated and euthanized in the course of other experiments [6], were collected at three different points of time presenting tissues from intact, OVX/untreated, and OVX/black cohosh-treated rats. Group 1 (PRAE) presented hippocampal (*n* = 4) and hypothalamic (*n* = 4) tissues from rats without OVX. Group 2 (OVX) presented hippocampal (*n* = 4) and hypothalamic (*n* = 3) tissues three months after OVX. Group 3 (OVX + iCR) presented hippocampal (*n* = 2) and hypothalamic (*n* = 2) tissues from rats after their treatment with a preparation of an isopropanolic extract from the rhizomes of *Cimicifuga racemosa* (iCR) (dry extract corresponding to 60 mg *Cimicifuga racemosa*/kg body weight; drug-extract-ratio 6–11:1; extraction solvent propan-2-ol [40% v/v]; Schaper and Brümmer, Germany) for three months after OVX. The sample material was stored at − 70 °C and − 80 °C until start of experiments. Animals with the same treatment and tissue were counted as replicates. All conditions of husbandry were in accordance with local regulations, and experimental procedures were approved and conducted under the auspices of a local German animal care and oversight committee [6].

### RNA preparation and sample processing

Total RNA was extracted from frozen hippocampus and hypothalamus tissue samples using QIAzol reagent followed by purification using an miRNeasy Mini kit (Qiagen, Hilden, Germany) and quantified using a Nanodrop UV–VIS spectrophotometer (Implen GmbH, München, Germany). RNA intactness and quality were confirmed using an Agilent 2100 Bioanalyzer (Wilmington, DE). The RNA-samples were stored at − 80 °C. Only samples with a RNA integrity number (RIN) greater than 8.0 were used for hybridization. All procedures were performed according to the manufacturer’s instructions.

### Gene expression microarray assays

100 ng of total RNA from each sample was labeled using the Low Input Quick Amp Labeling kit (Agilent Technologies Inc., Santa Clara, CA) following the manufacturer’s one-color microarray-based expression analysis protocol. RNA was then fragmented and hybridized to the 60-mer GeneExpression 4 × 44 K Rat Genome Arrays (Agilent Technologies Inc., Santa Clara, CA) for 17 h prior to washing and scanning. This microarray investigates 30,423 features (44 k is the theoretical maximum of dots on 44 k chips; many of them serve as internal controls or internal duplicates; this results in the current number of 30,423 available features).

Data were extracted from scanned images using the Agilent feature extraction software (version 11.5.1.1, Agilent Technologies Inc., Santa Clara, CA). The data processing of the arrays was carried out as follows: Differential gene expression analysis was performed setting contrasts between (i) PRAE and OVX, (ii) PRAE and OVX + iCR, and (iii) OVX and OVX + iCR for the hippocampus and hypothalamus, respectively. For cutoff thresholds, see below in statistics.

Gene ontology and pathway analyses were performed using the Broad Institute Gene Set Enrichment Analysis (GSEA) platform (19). GSEA is a computational method that determines whether an a priori defined set of genes shows statistically significant, concordant differences between two biological states (e.g., phenotypes) [7]. For visualization we used the”GOrilla” (Gene Ontology enRIchment anaLysis and visuaLizAtion) tool available at the GO-project [8]. This tool serves to discover GO terms that are significantly enriched at the top of a ranked gene list. Building on a complete theoretical characterization of the underlying distribution, GOrilla computes an exact p-value for the observed enrichment, taking threshold multiple testing into account. The output of the enrichment analysis is visualized as a hierarchical structure, providing a clear view of the relations between enriched GO terms [8]. The graphs show related GO terms in a tree structure highlighting enriched terms through a color code (strong enrichment in red).

### Quantitative polymerase chain reaction (qPCR)

#### Primer

Primers for qPCR reactions were selected using the “Primer design tool” by NCBI (https://www.ncbi.nlm.nih.gov/tools/primer-blast/). Selected target primer pairs used in the qPCR reaction are listed in Supplementary table 1.

#### qPCR procedure

Aliquots of the RNA, extracted and stored for the microarray, were used. The aim of the qPCR-analysis was to verify the expression value of each gene in the tissue samples. The residual RNA was insufficient to measure technical replicates in all 24 genes (22 target genes and 2 housekeeping genes). Therefore, the two biological replicates were used as the basis for statistical studies. cDNA was synthesized from 3 µg RNA by random priming using the Superscript II Reverse Transcriptase (Invitrogen, Carlsbad, CA). Transcript levels for targets found to be significantly up- or downregulated were measured in qPCR. Oligonucleotides were purchased from Biomers GmbH (Ulm, Germany). qPCR was performed with the LightCycler^®^ 480 Real-Time PCR System (Roche diagnostics, Risch, Switzerland) in a 20 µl reaction containing the SYBR Green based Luna Universal qPCR Master Mix (New England Biolabs, Frankfurt am Main, Deutschland), 250 nM forward primer, 250 nM reverse primer, SYBR and ROX in the concentrations provided by the manufacturer. Annealing and elongation temperature was 60 °C. Melting curve analysis of every qPCR was conducted resulting in sharp and single peaks. Efficiency of amplification was evaluated with wt [untreated prae probes]; the measured value was 100 ± 5%. Due to material restriction, all assays were performed without technical replicates. Ct values were determined using the second derivative method.

#### Relative gene expression calculation

The expression values were normalized to those of endogenous GAPDH and ACTB (housekeeping genes) using rat-specific primers. Relative expression was determined using the ΔΔCt method calculated with the appropriate LightCycler Software v1.5. With the ΔΔCt method, the expression value of the treated group (e.g., OVX or OVX + iCR) was expressed relative to the untreated control (PRAE) after normalization. The group “PRAE” was used as reference and set to “1”.

### Statistical analysis

The microarray data were extracted using Feature Extraction Software from Agilent Technologies (version 11.5). Subsequently, the extracted data were imported into the GeneSpring GX software (version 14.5), normalized (quantile normalization), and statistically analysed by predefined algorithms of the GeneSpring software, particularly ANOVA using the “Benjamini and Hochberg False Discovery Rate” method and threshold-selection. Statistical analysis is based on biological replicates (number of samples see 2.1). Primarily, cutoff was set for significantly (*p* value < 0.2) up- or downregulated genes that showed a fold change of at least 1.5-fold change (FC) in gene expression. Additionally, other threshold-combinations of the *p* value and the fold-change (FC > 3 at *p* < 0.5; any FC at *p* < 0.05; FC > 1.5 at *p* < 0.5) were investigated for reliability purposes.

The qPCR data were analysed for contrasts between the treatment groups using the *t* test.

## Results

### Microarray analysis

#### Selection, filtering, and compensation analysis of differentially expressed genes

Overall, 4812 genes were differentially regulated when applying ANOVA at *p* < 0.2 and FC > 1.5. Table 1 presents the number of differentially regulated genes when comparing the three groups – PRAE, OVX, and OVX + iCR – to each other within the respective brain tissue. Furthermore, compensating effects were observed. Compensation means that genes (i.e., features), where the contrast PRAE vs. OVX was at least 1.5-fold differentially expressed, were oppositionally regulated at the contrast OVX vs. OVX + iCR at least 1.5 times. This compensation analysis shows genes in which treatment with black cohosh (iCR) counteracted the effect of ovariectomy (OVX). The *p* value for the combinatory criterion was *p* < 0.04. The group of iCR-treated compensated features represented 213 genes in the hippocampus and 349 genes in the hypothalamus, thereof 44 features belonged to both tissue samples, resulting in 518 in total. In a next step, overlapping gene functions and regulatory KEGG pathways (https://www.genome.jp/kegg/pathway.html) were searched within each of the lists of these genes. For function and pathway analysis, a GSEA was carried out via the website “Gorilla” (http://cbl-gorilla.cs.technion.ac.il/). This tool arranges features that share the same function or participate in shared regulatory pathways [8]. The lists are output as a “function” for the gene ontology analysis and “process” for the pathway analysis. In addition, graphics are created for each analysis, which represent the strength of the enrichment of the respective processes or functions (example see Fig. 2). The focus of this analysis was on the OVX compensatory effects of iCR on 518 genes. It demonstrated the involvement of regulated genes in immune system-related processes, general signal transduction, aging, bone biology, body temperature-related processes, and hormone-related processes, particularly neuropeptide hormone activity and neuropeptide receptor binding (Supplementary Table 2).

**Table 1:** Number of differentially regulated genes

Hi Hy	PRAE	OVX	OVX + iCR	Compensation
PRAE		836	2401	213
OVX	943		2210	
OVX + iCR	1760	1759		
Compensation	349			

Selection, filtering, and compensation analysis of differentially regulated genes within the hippocampus and hypothalamus. The data are shown as a cross tabulation with the top right side showing the data for the hippocampus (Hi) and the bottom left side showing the data for the hypothalamus (Hy). The compensation analysis identified features in which treatment with black cohosh counteracted the effect of ovariectomy.

#### Exclusive effects of black cohosh on hippocampal and hypothalamic gene expression

**Fig. 1:**
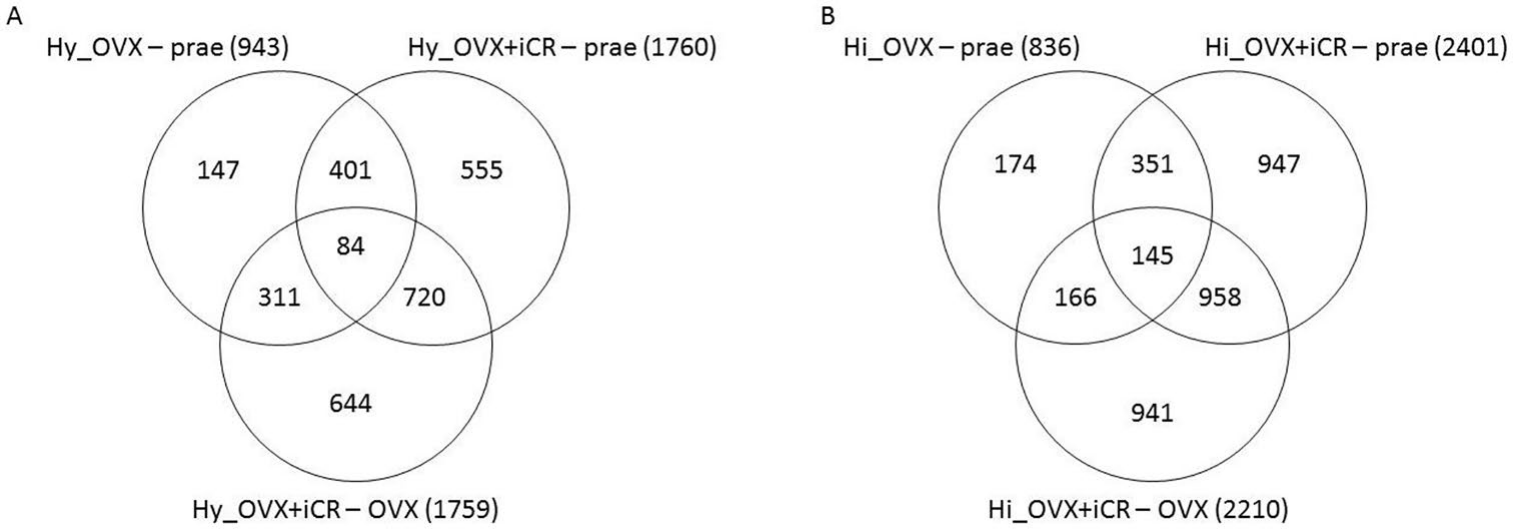
VENN diagram for differentially or overlappingly expressed genes in hippocampus and hypothalamus. The values used correspond to the features from Table 1, and they compare (**i**) PRAE vs. OVX, (**ii**) PRAE vs. OVX + iCR and (**iii**) OVX vs. OVX + iCR, each in hippocampus and hypothalamus. The *p* value cutoff was 0.2 in combination with the FC-threshold 1.5.

**Fig. 2:**
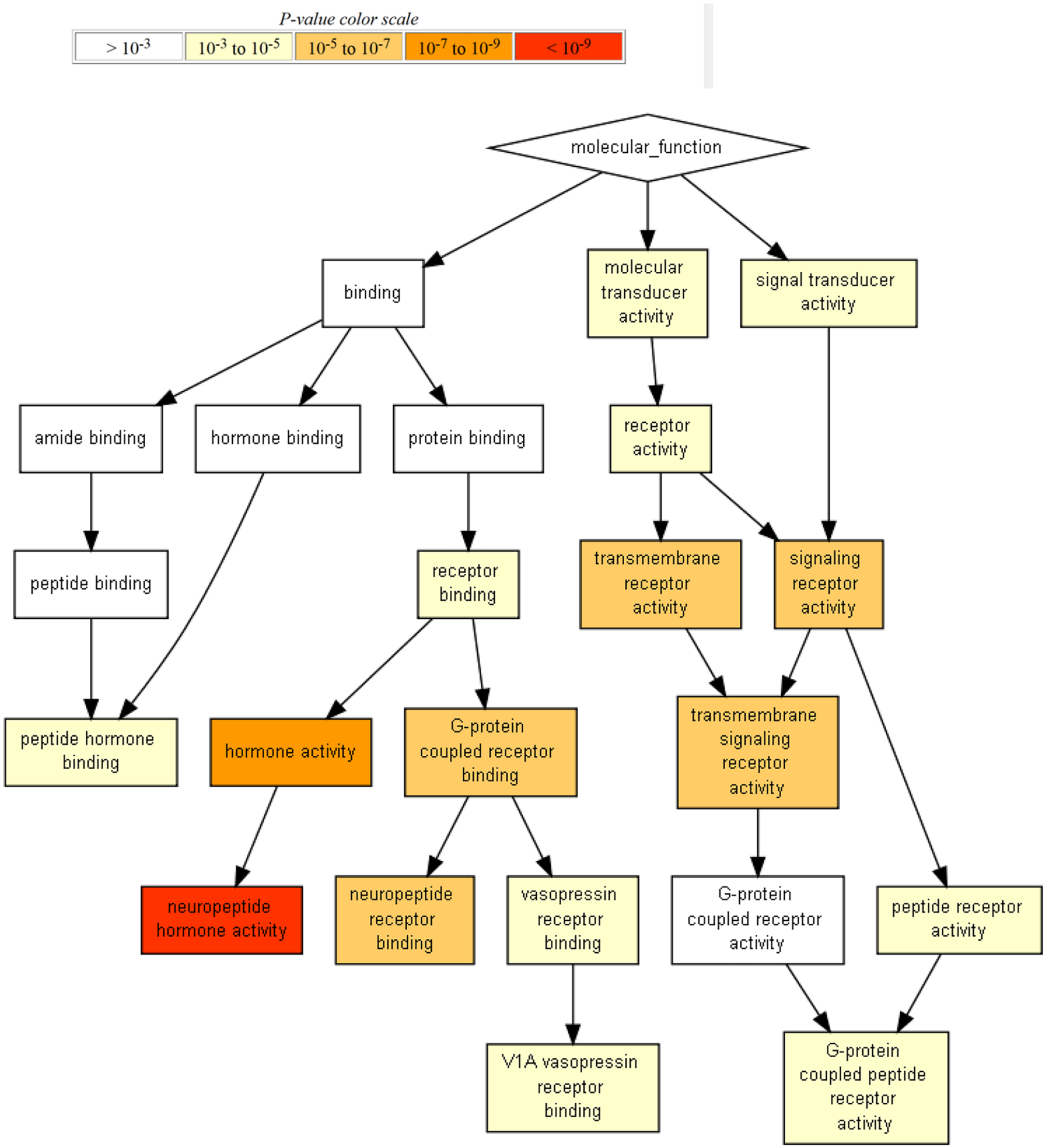
Example for a Gene Set Enrichment Analysis of the differentially expressed genes (compensation of OVX-effects by iCR) in the hippocampus (*n* = 213). The GOrilla tool identifies and visualizes enriched GO terms in ranked lists of genes. Genes whose OVX-induced change of expression is compensated by iCR treatment are ranked.

In order to estimate which genes were regulated in only one or two comparisons, an overlap analysis was performed. The aim was to detect genes in which the effect of one treatment (e.g., iCR) was not overlaid by another treatment (e.g., OVX) and could only be attributed to one single treatment (e.g., iCR). The focus of this analysis was on the exclusive effects of iCR. 644 genes in the hypothalamus and 941 genes in the hippocampus were assigned to these “iCR exclusive” clusters. Thereof, 42 features belonged to both tissue samples out of 1543 in total (Fig. 1). In a next step, we performed a GSEA for function and pathway analysis of the 1543 genes identified. It demonstrated the involvement of system classes like osmotic regulation, hormone-related activities, metabolism, and immune system (Supplementary table 3). Secondary analyses, with modulation of the fold change and the *p* value as described in statistics, confirmed the main outcomes of the GSEA of the primary analysis; immune system-related processes, general signal transduction, and hormone-related processes were the resulting pathways/terms in these modified analyses (Fig. 2).

### Target gene selection and qPCR expression values

In order to verify the results of the microarray-based functional analyses, 22 target genes were chosen for subsequent qPCR assays. As required, the housekeeping genes were not differentially expressed by any treatment group in the microarray. The expression values of these two genes were in the range of the other microarray-assayed reference genes. The pathways of interest resulted by the GSEA analysis “compensation” and “iCR exclusive” (see above) were screened for specific target genes that best represented the resulting pathways. Additionally, further target genes were chosen due to their relevance in scientific discussions even though these genes were not featured within the microarray and GSEA analyses. Genes from the olfactory receptor (OR) gene family were already covered by previous analyses [9]; therefore, they were not considered for renewed qPCR verification. Taken together, these three different core issues yielded the following candidate gene categories: (1) target gene selection based on “compensation analysis” (FC > 1.5 at *p* < 0.2; filter: compensation), (2) target gene selection based on”GSEA iCR exclusive” (FC > 1.5 at *p* < 0.2, filter: iCR exclusive) not covered by”compensation analysis”, and (3) generally important target genes not included in the above mentioned lists (Table 2, Supplementary table 4).

**Table 2:** Selected target genes for qPCR

No	Gene	Name
1. Target gene selection based on Table”GSEA compensation analysis “ (FC > 1.5, *p* < 0.2; Filter: compensation). Question: Which OVX-induced changes are compensated by iCR?		
1	AVPR1A (Hi)	Arginine vasopressin receptor 1A
2	GAL (Hi)	Galanin and GMAP prepropeptide
3	CALCA (Hi)	Calcitonin related polypeptide alpha
4	HCRT (Hi)	Hypocretin neuropeptide precursor
5	PNOC (Hi)	Prepronociceptin
6	IL5 (Hi)	Interleukin 5
7	BCL2 (Hy)	BCL2, apoptosis regulator
8	IGFBP1 (Hy)	Insulin like growth factor binding protein 1
9	IGFBP5 (Hy)	Insulin like growth factor binding protein 5
10	IL3 (Hy)	Interleukin 3
11	TRPV3 (Hy)	Transient receptor potential cation channel subfamily V member 3
2. Target gene selection based on "GSEA iCR exclusive “ (FC > 1.5, *p* < 0.2; Filter: iCR exclusive). Question: Which gene expression is exclusively changed by iCR?		
12	PRL7A3 (Hi)	Prolactin family 7, subfamily a, member 3
13	PLCB1 (Hy)	Phospholipase C beta 1
14	MAPK9 (Hy)	Mitogen-activated protein kinase 9
15	ADCY1 (Hy)	Adenylate cyclase 1
16	G6PC (Hy)	Glucose-6-phosphatase catalytic subunit
17	CACNA1A (Hy)	Calcium voltage-gated channel subunit alpha1 A
3. Generally important target genes not covered in above mentioned lists. Question: Which further genes are of general importance in regard to the present scientific work?		
18	ESR1	Estrogen receptor 1 (alpha)
19	ESR2	Estrogen receptor 2 (beta)
20	OPRM1	Opioid receptor mu 1
21	KISS1	KiSS-1 metastasis-suppressor
22	TAC3	Tachykinin 3

Overview of selected target genes. Hi = Hippocampus, Hy = Hypothalamus, OVX = bilateral ovariectomy, iCR = isopropanolic extract of Cimicifuga racemosa (black cohosh).

Relative qPCR expression values are presented in Table 3. Group comparisons of gene expression levels evaluated by qPCR were assessed regarding a combination of fold-change and significance (Table 4) and assessed related to the microarray results.

**Table 3:** qPCR values expressed as fold change, and intergroup comparisons (*t* test).** a** hippocampus,** b** hypothalamus

a																							
Hippocampus		AVPR1A	GAL	CALCA	HCRT	PNOC	IL5	BCL2	IGFBP1	IGFBP5	IL3	TRPV3	PRL7A3	PLCB1	MAPK9	ADCY1	G6PC	CACNA1A	ESR1	ESR2	OPRM1	KISS1	TAC3
Fold change	PRAE	1.000	1.000	1.000	1.000	1.000	1.000	1.000	1.000	1.000	1.000	1.000	1.000	1.000	1.000	1.000	1.000	1.000	1.000	1.000	1.000	1.000	1.000
	OVX	1.125	1.653	1.434	2.780	1.206	0.622	1.591	1.117	0.959	0.832	1.149	1.087	1.181	1.223	0.707	2.056	0.946	1.564	1.586	1.017	1.046	1.429
	OVX + iCR	0.468	0.927	0.676	0.572	0.543	0.159	1.227	0.657	0.644	0.530	0.611	0.493	0.642	0.555	0.511	0.722	0.920	0.838	0.949	0.920	0.483	0.637
Inbetween group comparisons	PRAE-OVX	0.270	0.001	0.087	0.135	0.190	0.000	0.011	0.502	0.607	0.073	0.024	0.034	0.070	0.090	0.005	0.016	0.065	0.016	0.050	0.812	0.359	0.011
	OVX-(OVX + iCR)	0.029	0.021	0.069	0.128	0.026	0.009	0.055	0.029	0.004	0.002	0.002	0.003	0.003	0.005	0.001	0.033	0.995	0.039	0.076	0.705	0.005	0.006
	PRAE-(OVX + iCR)	0.002	0.458	0.046	0.099	0.000	0.002	0.220	0.117	0.026	0.016	0.016	0.001	0.003	0.003	0.001	0.125	0.734	0.158	0.409	0.976	0.023	0.002

b																							
Hypothalamus		AVPR1A	GAL	CALCA	HCRT	PNOC	IL5	BCL2	IGFBP1	IGFBP5	IL3	TRPV3	PRL7A3	PLCB1	MAPK9	ADCY1	G6PC	CACNA1A	ESR1	ESR2	OPRM1	KISS1	TAC3
Fold change	PRAE	1.000	1.000	1.000	1.000	1.000	1.000	1.000	1.000	1.000	1.000	1.000	1.000	1.000	1.000	1.000	1.000	1.000	1.000	1.000	1.000	1.000	1.000
	OVX	1.233	0.974	0.890	0.783	0.928	1.033	0.878	0.721	0.682	0.647	1.299	0.884	1.070	1.540	0.961	1.487	0.791	1.179	0.922	0.900	2.096	1.131
	OVX + iCR	0.759	0.696	0.691	0.492	0.610	3.199	0.645	0.762	0.691	0.897	0.991	0.968	0.822	1.055	0.632	1.834	0.492	0.713	0.854	0.600	2.383	0.875
Inbetween group comparisons	PRAE-OVX	0.001	0.370	0.104	0.172	0.281	0.325	0.139	0.002	0.025	0.110	0.009	0.026	0.400	0.001	0.604	0.143	0.027	0.031	0.007	0.034	0.019	0.024
	OVX-(OVX + iCR)	0.035	0.151	0.081	0.013	0.037	0.178	0.017	0.524	0.460	0.047	0.064	0.348	0.094	0.046	0.025	0.290	0.007	0.038	0.002	0.001	0.009	0.080
	PRAE-(OVX + iCR)	0.147	0.172	0.089	0.025	0.073	0.180	0.005	0.048	0.037	0.285	0.970	0.838	0.403	0.504	0.085	n.a	0.001	0.161	0.001	0.001	0.016	0.351

qPCR values expressed as fold change and intergroup comparisons (t-test). a) hippocampus, b) hypothalamus; PRAE = intact animals, OVX = bilateral ovariectomy, OVX+iCR = treatment with isopropanolic extract of Cimicifuga racemosa (black cohosh) after OVX.

**Table 4:** Comparison of microarray and qPCR results

Gene	Question for selecting this gene (details see text)	Hippocampus	Hypothalamus
AVPR1A	Compensation (Hi)	#^2^	XX^3^
GAL	Compensation (Hi)	XX	O
CALCA	Compensation (Hi)	XX	O
HCRT	Compensation (Hi)	XX	X
PNOC	Compensation (Hi)	#^2^	< >
IL5	Compensation (Hi)	< >	< >
BCL2	Compensation (Hy)	< >	< >
IGFBP1	Compensation (Hy)	xx^3^	< >
IGFBP5	Compensation (Hy)	< >	< >
IL3	Compensation (Hy)	< >	< >
TRPV3	Compensation (Hy)	< >	< >
PRL7A3	iCR exclusive (Hi)	< >	XX^3^
PLCB1	iCR exclusive (Hy)	< >	< >
MAPK9	iCR exclusive (Hy)	< >	< >
ADCY1	iCR exclusive (Hy)	< >	< >
G6PC	iCR exclusive (Hy)	< >	< >
CACNA1A	iCR exclusive (Hy)	< >	< >
ESR1	General interest	#^1^	#^1^
ESR2	General interest	#^1^	O
OPRM1	General interest	O	#^2^
KISS1	General interest	< >	O
TAC3	General interest	#^1^	XX^3^

“XX” = qPCR and microarray mutually show ‘compensation of OVX-induced effect by iCR’

“X” = qPCR and microarray mutually show ‘exclusive effect of iCR’

“O” = qPCR and microarray show ‘no relevant effect of iCR’

“<>”= qPCR and microarray results are not consistent with each other

“#” = target genes, which do not show similar patterns at the two techniques but are worth to be discussed

1 compensation in qPCR, but no such effect found in the microarray

2 compensation in microarray, but in qPCR only a trend of an OVX-effect and a significant iCR-effect was found

3 compensation in qPCR, but in microarray only a trend was seen which did not meet the FC threshold criteria.

### Comparison of microarray with qPCR results

Individual patterns were used to compare the expression of the 22 selected target genes between microarray and qPCR analysis: Equivalent to the qPCR results, array values were normalized and the tissue “PRAE” was set to “1”. The fold changes of OVX and OVX + iCR in both assays were compared directly and assessed together (exemplary diagrams see Supplementary Fig. 1). In Table 4 the genes are marked where a match exists. The match/non-match classification was driven by the queries “compensation”, “iCR exclusive” or “general interest”. Regarding qPCR, the criteria for “compensation” were: qPCR-values of PRAE and OVX + iCR were both lower or both higher than the qPCR-value of OVX, and the fold-change PRAE versus OVX or OVX versus OVX + iCR, respectively, was either > 1.5 or < 1/1.5 or significant at *p* < 0.05.

Overall, some target genes were confirmed (10 in 44) with the verification technique qPCR. GAL and CALCA were verified best. The expression of HCRT was only partially confirmed due to the high standard deviations in both methods. Due to low signal strength in the arrays of the genes IL3, IL5, and PRL7A3, the missing concordance of both assays was expectable. If genes had a strong expression level and good signal strength in the microarray, both methods revealed similar expression patterns.

Taken together, predominantly the compensatory effect of iCR within the hippocampus tissue was confirmed by qPCR. Particularly the genes GAL, CALCA, and HCRT showed good consistency. The results of the genes AVPR1A and PNOC were less consistent. Here the compensatory effect was found in the microarray, but in the qPCR, only a trend of an OVX-induced increase of the gene expression level was found in comparison to PRAE. However, the iCR-induced decrease was still significant indicating a compensation. ESR1, ESR2, and TAC3 did not meet the threshold criteria in the microarray, but in the qPCR, a compensatory effect was found.

## Discussion

The microarray analyses revealed different expression profiles in the brain sections from ovariectomized (OVX) or iCR-treated rats after ovariectomy (OVX + iCR) compared to intact and untreated rats (PRAE). 3682 features out of > 30,000 in the hippocampus and 2862 features in the hypothalamus were significantly up- or downregulated. Thereof, 1732 features belonged to both tissue types resulting in 4812 in total. Comparing OVX and OVX + iCR, 2210 genes in the hippocampus and 1759 in the hypothalamus were identified as differentially expressed, i.e., resembled iCR-effects in tissues of ovariectomized rats.

Three approaches were used to discover relevant pathways and target genes in menopausal transition and iCR treatment: ‘compensation’, ‘iCR exclusive effects’, and those of ‘general’ interest. 213 genes in the hippocampus and 349 genes in the hypothalamus, counted in the compensation approach, were identified by testing them for contrary fold change. The cluster with 941 genes in the hippocampus and 644 genes in the hypothalamus displayed candidates of an exclusive iCR effect, i.e., in genes not modulated by ovariectomy. GSEA analyses on 1543 genes that were not changed by OVX revealed that black cohosh affects pathways involved in system classes such as osmotic regulation, hormone-related activities, metabolism, and immune system. Next to the contribution of plenty of OR family genes in signalling pathways, which were already covered in previous analyses [9], some additional pathways also appeared to specifically contribute during the menopausal transition and the abolishment by iCR. Black cohosh compensated the effects of OVX in 518 genes, which were allocated by GSEA analysis to following the processes and systems, respectively: immune system, general signal transduction, aging, bone biology, body temperature, and hormone-related processes, particularly neuropeptide hormone activity and neuropeptide receptor binding.

In order to verify the results of microarray and functional analyses with a second technique, target genes were chosen for subsequent qPCR assays. In respect to the first two approaches mentioned above, the picked pathways of interest from the two lists GSEA “compensation” and GSEA “iCR exclusive” were screened for specific target genes that best present the respective pathways. In addition to the 17 target genes thus selected, five further target genes were chosen due to scientific discussions [10–19] even though the criteria for approach 1 and 2 were not met. The 22 target genes were only few of the many genes tested on the 30,423-feature microarray. Unfortunately, the available RNA was restricted, so no more target genes could be assayed by qPCR. Nevertheless, due to plenty of overlapping functions, the whole panel of pathways of interest was represented by these 22 target genes. Thus, the selected target genes were not a random selection. An aim of this study was to find genes possibly targeted by black cohosh and to investigate theories about its mode of action. The procedures were chosen to maximize the gain in knowledge about iCR’s influence on brain function.

The expression values of the residual RNA were not sufficient enough to measure technical replicates. Therefore, the two biological replicates were used as the basis of statistical studies. This procedure differed in the qPCR from the microarray analysis; therefore, the values may partially differ resulting in some source of error-proneness. Nevertheless, the averages of both samples and the inference of the results of both methods (microarray and qPCR) are decisive. A major limitation of this study is the low sample size; the interpretation should be tentative. Additional studies may confirm these results with more impact.

The concordance of the results was assessed in regard to the selection criteria: compensating or iCR exclusive effect on the hippocampus or hypothalamus, or general interest in the gene. Statistical analysis was not reasonable for this assessment; a graphical comparison sufficed. All in all, predominantly compensation at the hippocampus was confirmed by qPCR. Particularly GAL, CALCA, and HCRT showed good consistency. The results of the genes AVPR1A and PNOC were somewhat less consistent showing compensation in the microarray but only a trend of an OVX-induced increase of the expression value in comparison to PRAE in the qPCR. However, the iCR-induced decrease was still significant (Supplementary Fig. 1). Thus, if OVX increases the expression of these genes, iCR can compensate such effect in hippocampus. This effect might alternatively be interpreted as over-compensatory with residual uncertainty as to which interpretation holds true regarding these two genes in this tissue. The signal intensity of IL5 was very low; therefore, the missing concordance with the qPCR results was expectable. Apart from IL5, all of the selected target genes in the hippocampal compensation query were more or less confirmed.

The compensation at the hypothalamus and all iCR exclusive effects found in the microarray analysis were not confirmed by the qPCR in the tissue of primary focus, e.g., this concerns pathways regarding metabolism and pathways regarding the immune system (Table 4). Unexpectedly, the pattern in the PCR sometimes gave a mirror image of that in the microarray, meaning an effect was obvious but in the other direction, e.g., hypothalamus PLCB1, MAPK9, TRPV3. This finding cannot be adequately explained. Limited sample size, sample material, and signal intensities may contribute to these circumstances.

As a matter of completeness, the expression of the selected genes was also investigated by qPCR in the other tissue. Regarding AVPR1A, GAL, CALCA, HCRT, and PNOC, the pattern of effects or no effects on gene expression in the hypothalamus differed from the clear pattern in the hippocampus (Table 4). Interestingly, IGFBP1 in the hippocampus and PRL7A3 in the hypothalamus showed a compensation pattern in the qPCR of the other tissue with only a trend in the microarray, which did not meet the FC-threshold criteria.

The compensating effect of iCR was found by qPCR also for the genes ESR1, ESR2, and TAC3 in the hippocampus even though there was no such effect in the microarray. The patterns of TAC3 at the hypothalamus were similar in both methods; a compensational effect was observed. This gene was not filtered by the selection procedure of the microarray results, because the 1.5-fold change was not attained (fold change OVX vs. OVX + iCR =  − 1.32). Furthermore, the KISS1 microarray pattern of the hypothalamic sample was confirmed. OVX and OVX + iCR showed higher expression values than PRAE; an OVX effect was shown. For OPRM1 the qPCR confirmed no relevant effect of OVX or iCR on OPRM1 expression in the hippocampus. In the hypothalamus, the microarray’s *p* value results for OPRM1 might indicate a compensation of the OVX-induced decrease by iCR. However, this OPRM1 result did not meet the FC-threshold-criteria, and in the qPCR, only a trend of an OVX-effect accompanied a significant iCR-effect.

Since the target region of the genes through the qPCR is larger (80–129 bp, mean 108.8 bp) than in the 60-mer microarray, the specificity of the technique is enlarged too. On the other hand, the sample number was higher for the microarray. Therefore, finding a balance between the results from both techniques is difficult. In general, the results of the two techniques match well [20], but this depends on filtering parameters etc. [21]. The absolute Ct values or signal intensities should also be taken into account when assessing the concordance of the results. The most critical factors influencing the correlation between microarrays and qPCR are fold change and *p* value values of the microarray, but Ct values and up- vs. downregulation are also important [21]. The match of the transcripts in both methods also plays a role in the agreement of the results [22]. Even if the qPCR transcripts are as close as possible to the corresponding microarray features, there are, in principle, certain differences in the targeted gene region yielding different results.

Although microarray analyses revealed compensation by iCR in the hippocampus and qPCR even revealed overcompensation of some genes (AVPR1A, PNOC), this result should not be overestimated as the number of samples was too small to reach a final conclusion. In addition, statistical analyses solely for significance should be interpreted cautiously as the sample size was not appropriate for such a test. A combination of the thresholds for FC and p-value is more informative. This study gives hints to help identify the underlying mechanisms after OVX and the mode of action of iCR. Additional studies may be done to follow-up on this information.

However, system processes are not only regulated by changed gene expression. Posttranslational protein modifications and elevated depletion or stability of the gene product also contribute to cell regulation. In such cases, protein analyses should then also be performed.

The GSEA of hippocampal samples resulted in some compensated pathways/terms of major interest within the transformation process during menopause as follows: positive regulation of blood pressure, hormones, feeding behaviour, cytokine-cytokine receptor interaction, inflammatory response, response to insulin, orexigenic neuropeptide QRFP/P518, opioid receptor binding, cytokine activity, and positive regulation of vasoconstriction. The good concordance of the target gene analyses by PCR with the microarray results in the hippocampal compensation approach leads to the conclusion that the GSEA obtained pathways/terms are also confirmed. From these analyses, it cannot be determined whether there is one single receptor for iCR or in which order the downstream processes happen; only involvement is shown. All in all, some signalling paths in brain performance are altered due to ovariectomy and several of these changes are restored by iCR in the hippocampus.

Of course, ovariectomy also results in more widespread effects and is not completely identical to the processes that occur during menopausal transformation. Nevertheless, studies with this animal model have provided hints towards a better understanding of the CNS processes caused by oestrogen decline and of the mode of action of *Cimicifuga racemosa*.

The analysis of the mode of action for CR is very difficult because the multicomponent herbal preparation—the extract—performs many different individual actions. As previously hypothesized, there is a multi-target mode of action and the resulting effect is that CR restores imbalanced functions and mitigates menopausal symptoms by a multifaceted mechanism [23, 24].

Gaube et al. [25] investigated the effects of a lipophilic black cohosh rhizome extract, E2, and tamoxifen on the oestrogen receptor positive human breast cancer cell line MCF-7 using an Affymetrix GeneChip^®^ Human Genome array. A wide range of cellular pathways and targets were affected by black cohosh. Most of the regulated genes could be clearly assigned to five larger groups of functionally related genes: apoptosis, proliferation, general growth, signalling and transport, and metabolism. Previously reported actions of black cohosh were confirmed by gene expression assigned by “contra cell proliferation” and “pro apoptosis”. The action of black cohosh in MCF-7 cells seems to be neither oestrogenic nor antiestrogenic but rather multifaceted. The analyses presented in this study do not accord with that of Gaube et al. [25] in detail, probably due to the different sample material and study design, in particular (in vitro/in vivo; cancer cell line/healthy primary samples; treatment only/treatment after OVX).

Beginning with 30,423 features in the microarray, a reduction to a few individual genes with a contribution to concise signalling pathways was achieved in the course of all the analyses. iCR can compensate OVX-induced changes/hypoestrogenism in the brain, particularly the hippocampus. This is reflected by changes in the expression profiles of genes that not only contribute to the formation of hot flushes or central thermoregulation but also to secondary effects such as blood pressure, metabolism, nociception, hormonal regulation, homeostasis, cognition, mood regulation, neuroendocrine modulation, peripheral thermoregulation through altered blood flow at the cutaneous microvascular level, regulation of sleep and arousal, learning, and memory. These relationships to processes in the menopause deserve further clarification.

## Conclusion

This set of exploratory qPCR and microarray analyses of rat hippocampus and hypothalamus tissue samples reveals effects of iCR on OVX-induced changes in the expression of genes. Some signalling paths are changed due to ovariectomy and subsequent oestrogen decline, and this altered brain performance is, at least partially, restored by iCR in the hippocampus. These paths include regulation of blood pressure, metabolism, hormonal regulation, homeostasis, cognition, mood regulation, neuroendocrine modulation, thermoregulation through altered blood flow at the cutaneous microvascular level, regulation of sleep and arousal, learning, and memory. All in all, this pilot study contributes to the evidence on the multifaceted mode of action of iCR in alleviating menopausal complaints on a tissue-specific CNS gene expression level.

## Supplementary Information

Below is the link to the electronic supplementary material.

Supplementary file1 (DOCX 334 KB)

## Data Availability

The authors confirm that the data supporting the findings of this study are available within the article and its supplementary materials.

## Abbreviations

FC: Fold Change
GOrilla: Gene Ontology enRIchment anaLysis and visuaLizAtion
GSEA: Gene Set Enrichment Analysis
Hi: Hippocampus
Hy: Hypothalamus
iCR: Isopropanolic extract of *Cimicifuga racemosa* rhizome
OR: Olfactory receptor
OVX: Bilateral ovariectomy
OVX + iCR: Treatment with iCR after OVX
PRAE: Tissues of rats without OVX
qPCR: Quantitative (reverse transcription) Polymerase Chain Reaction
