# Supplementary material for: Gene expression analyses on multi-target mode of action of black cohosh in menopausal complaints – a pilot study in rodents
Source: Arch Gynecol Obstet. 2021 Jul 14;305(1):275–86. doi: 10.1007/s00404-021-06105-8 (PMC8782806; doi:10.1007/s00404-021-06105-8)
Supplement: Supplementary file 1 — Supplementary file1 (DOCX 334 KB) [file 404_2021_6105_MOESM1_ESM.docx]

Supplementary Figure 1: Comparison of normalized qPCR and array data: Normalized qPCR data (left, fold change) and normalized microarray data (right, fold change) are shown in separate diagrams. PRAE samples were set at “1”. The p-values (t-test for intergroup comparisons) of the indicated comparisons are recorded in the row below the columns. The signal intensities of the microarrays are inserted in the right diagram. Each target gene is shown separately. a) AVPR1A, b) GAL, c) CALCA, d) HCRT, e) PNOC, f) TAC3, g) ESR1, h) ESR2.

a)


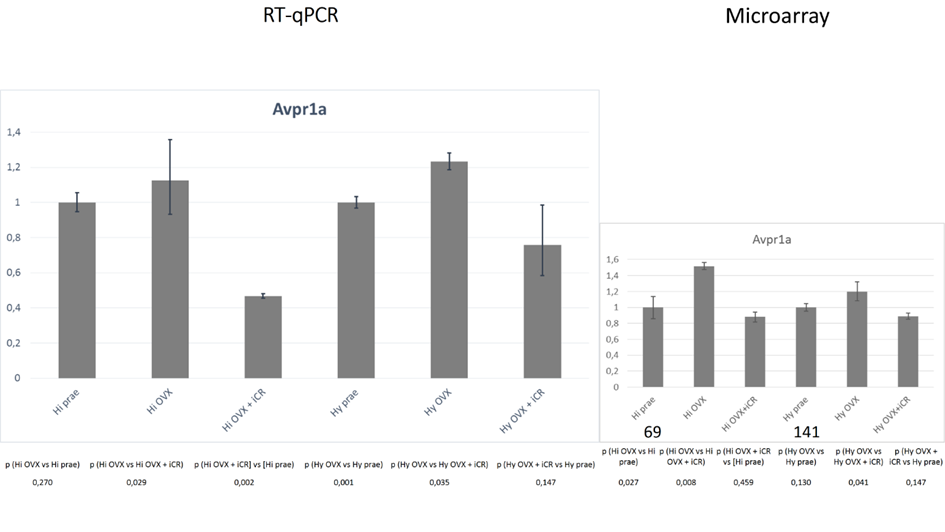


b)


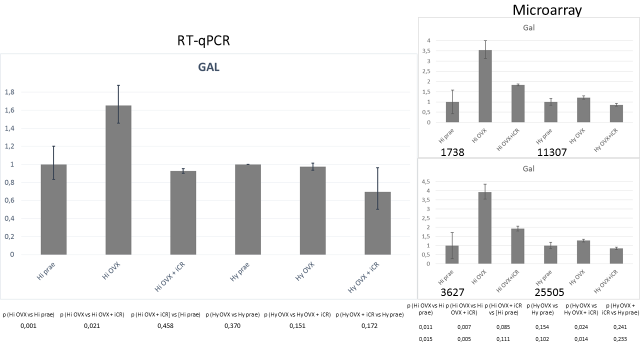


c)


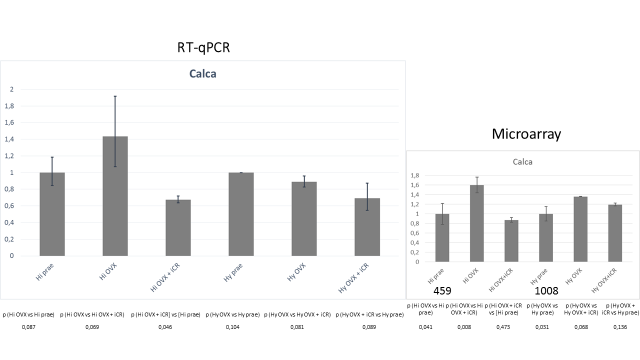


d)


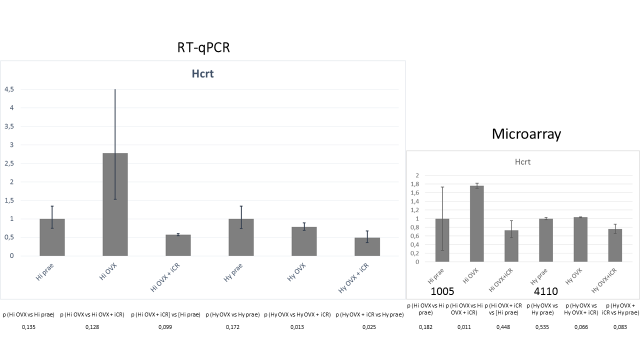


e)


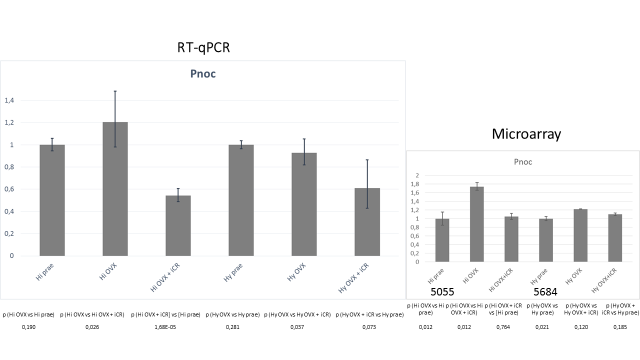


f)


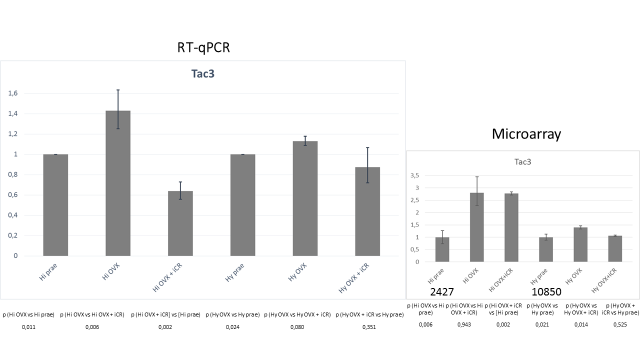


g)


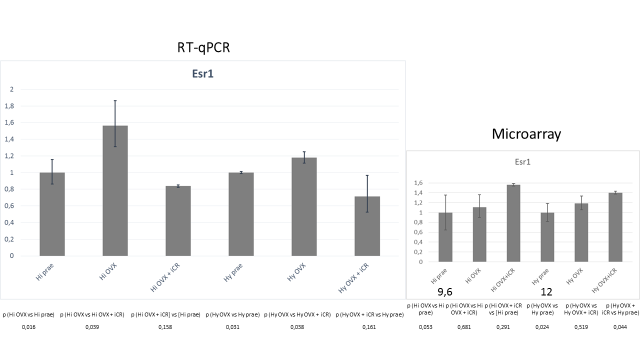


h)


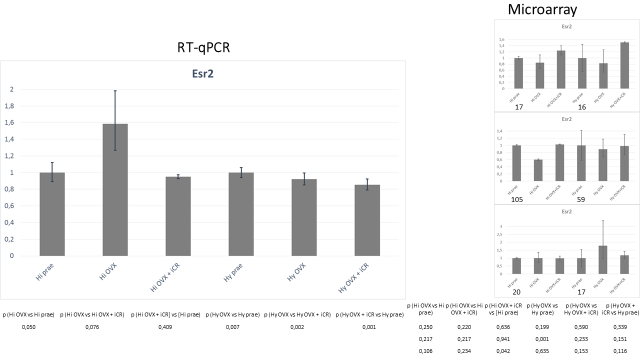


Supplementary Figure 1

Supplementary table 1: Used primers for qPCR verification.

Supplementary table 2: Excerpt of the GSEA analysis for samples whose OVX-induced change of expression was compensated by iCR treatment (213 hippocampus and 349 hypothalamic samples). The p-values denote the error probability of the respective GSEA-result regarding the cluster of genes included to the respective term.

Supplementary table 3: Excerpt of the GSEA analysis for samples whose expression was exclusively caused by iCR treatment after OVX (941 hippocampus and 644 hypothalamic features). The p-values denote the error probability of the respective GSEA-result regarding the cluster of genes included to the respective term.

Supplementary table 4: Overview of selected target genes (gene code, full gene name, description/relevance based on NCBI “gene” database (<https://www.ncbi.nlm.nih.gov/gene/)>).

Supplementary table 1

| **Name fw** | **Sequence fw** | **Name rv** | **Sequence rv** |
| --- | --- | --- | --- |
| AVPR1A 1148F | GTAAGCGCCTACATCCTTTGC | AVPR1A 1259R | CCGTGATTGTGATGGAAGGG |
| GAL 390F | GCCTGAGAGCAATATCGTCC | GAL 509R | TCTAGGTCTTCTGAGGAGGTGG |
| CALCA 502F | GGGACCTTCAGGCTTGAACAG | CALCA 619R | GTGTCTTCCATCAGCCTTTCTG |
| HCRT 64F | CCTCTCCCTGAGCTCCAGAC | HCRT 180R | CGTCCACCCCAAGCGACAGC |
| PNOC 562F | AATGTGTAGCCAGAAGGAGCC | PNOC 658R | GTCTTGGTGTGGACACATGC |
| IL5 140F | CGATGAGGCTTCCTGTTCCTA | IL5 253R | GTATTTCCACAGTGCCCCCTC |
| BCL2 752F | GGATGACTGAGTACCTGAACCG | BCL2 868R | GCCAGGAGAAATCAAACAGAGG |
| IGFBP1 805F | CTGCCAAACTGCAACAAGAATG | IGFBP1 904R | CACTCCATGGGTAGACACACC |
| IGFBP5 854F | GAAAAGAGCTACGGCGAGCAA | IGFBP5 960R | CGGAAGACCTTCGGGGAGTAG |
| IL3 190F | ATCCTCAGGTATCTGGACTCAA | IL3 269R | GTCCAGGTTTACTCTCCGCAA |
| TRPV3 1785F | GCTTCCAGTCTATGGGCATGT | TRPV3 1899R | GCCAGCGCTACTCCAAATCC |
| PRL7A3 510F | GCTGAGCTTTTCGAGTACACC | PRL7A3 596R | AAGACCAGAAAAGATGGAGCGA |
| PLCB1 3590F | AAACACAAGGAGATCCGCCA | PLCB1 3692R | TCTCCAGGGGCAGTCTTTTG |
| MAPK9 905F | GGGAGAGCTGGTGAAAGGTT | MAPK9 1005R | TGAACTCTGCGGATGGTGTT |
| ADCY1 2864F | TCAATGTGGCCAGTCGGATG | ADCY1 2952R | TGAGCACCTGTTCAGCAGTC |
| G6PC 565F | TGTCTGTCCCGGATCTACCTT | G6PC 676R | CATTGTAGATGCCCCGGATGT |
| CACNA1A 5331F | CCACCACCTGGATGAGTACG | CACNA1A 5443R | CGAGAGGGGGCGATATTACT |
| ESR1 1546F | AGGGAGAAGAGTTTGTGTGCC | ESR1 1668R | TCTTGTCCAGGACTCGGTGG |
| ESR2 1667F | TTCTCACGTCAGGCACATCAG | ESR2 1795R | GACTTGTACCCTCGAAGCGTG |
| OPRM1 447F | AGTGGGCCTCTTCGGAAACT | OPRM1 568R | GTGTACTGGTCGCTAAGGCG |
| KISS1 34F | CTCCTCTGTGTGGCCTCTTTT | KISS1 135R | ATTAACGAGTTCCTGGGGTCC |
| TAC3 151F | CTCAGCTTGGCATGGACCTT | TAC3 266R | TAGAGTCTCCGAAGCAGGGG |
| GAPDH F | GTGCCAGCCTCGTCTCATAG | GAPDH R | AGAGAAGGCACCCTGGTAA |
| ACTB F | CTAAGGCCAACCGTGAAAAGATG | ACTB R | GTGGTACGACCAGAGGCATAC |

Supplementary table 2

| **Hippocampus** |  |  |  |  |
| --- | --- | --- | --- | --- |
| **Category** | **Term** | **Count** | **PValue^[[1]](#footnote-1)^** | **Genes** |
| GOTERM_MF_DIRECT | GO:0005184~neuropeptide hormone activity | 9 | 7.54E-11 | CALCA, HCRT, AVP, PNOC, CARTPT, GAL, QRFP, NPPA |
| GOTERM_BP_DIRECT | GO:0007218~neuropeptide signalling pathway | 11 | 3.46E-09 | CALCA, HCRT, PNOC, PMCH, NPFFR2, CARTPT, GAL, QRFP, NPPA, GLP1R |
| UP_KEYWORDS | Neuropeptide | 7 | 5.47E-08 | HCRT, PNOC, PMCH, CARTPT, GAL, QRFP |
| UP_SEQ_FEATURE | signal peptide | 32 | 8.92E-07 | CALCR, HCRT, ARSI, TNFRSF8, KITLG, CALCA, FGA, RLN1, GPX5, IL10RA, PRSS3, CES1D, FAM150B, QRFP, AVP, IL5, PMCH, PRL8A9, GAL, EPHA7, ITPRIPL1, PNOC, CDH17, PRPMP5, GRM6, CARTPT, CHRND, TSHR, IGSF9, CSN3, GLP1R, NPPA |
| GOTERM_BP_DIRECT | GO:0045777~positive regulation of blood pressure | 6 | 5.63E-06 | AGTR1A, AVPR1A, CARTPT, QRFP, GLP1R |
| UP_KEYWORDS | Hormone | 7 | 1,30E-04 | CALCA, AVP, RLN1, PMCH, PRL8A9, GAL, NPPA |
| GOTERM_BP_DIRECT | GO:0007631~feeding behavior | 5 | 1.42E-04 | CALCA, HCRT, PMCH, GAL, GLP1R |
| GOTERM_BP_DIRECT | GO:0051970~negative regulation of transmission of nerve impulse | 3 | 5.71E-04 | HCRT, AVP, AVPR1A |
| KEGG_PATHWAY | rno04060:Cytokine-cytokine receptor interaction | 7 | 0.00398 | IL5, IL10RA, TNFRSF18, KITLG, TNFRSF8, TNFSF14, IFNLR1 |
| GOTERM_BP_DIRECT | GO:0006954~inflammatory response | 8 | 0.00787 | CALCA, CCL1, IL5, AGTR1A, TNFRSF18, SCN9A, TNFRSF8, GAL |
| GOTERM_BP_DIRECT | GO:0002125~maternal aggressive behavior | 2 | 0.0302 | AVP, AVPR1A |
| GOTERM_BP_DIRECT | GO:0032870~cellular response to hormone stimulus | 3 | 0.0770 | UCP3, NPFFR2, AVPR1A |
| **Hypothalamus** |  |  |  |  |
| **Category** | **Term** | **Count** | **PValue** | **Genes** |
| UP_SEQ_FEATURE | signal peptide | 24 | 7.11E-04 | FMOD, IL3, AEBP1, LTBP2, OSMR, GLRA3, TNFRSF8, FGF10, IGF2, SDC4, CPZ, OMD, INHBE, PRSS3, SERPINB2, CHRNB3, WFDC15A, DEFB43, MADCAM1, IGFBP1, LBP, FAM150B, RNASE13, IGFBP5 |
| GOTERM_BP_DIRECT | GO:0050911~detection of chemical stimulus involved in sensory perception of smell | 25 | 0.0102 | OLR1016, OLR1305, OLR1351, OLR1302, OLR159, OLR98, OLR650, OLR558, OLR1423, OLR703, OLR1413, OLR1024, OLR1630, OLR510, OLR1321, OLR1660, OLR1681, OLR1765, LOC684471, OLR689, OLR1206, OLR1654, OLR921, OLR1232, OLR852 |
| GOTERM_BP_DIRECT | GO:0006955~immune response | 8 | 0.0210 | IL3, C7, TNFRSF11A, TNFRSF8, TNFSF14, LOC691670, CCR1L1, NKX2-3 |
| GOTERM_BP_DIRECT | GO:0006006~glucose metabolic process | 4 | 0.0308 | IGF2, IGFBP1, TCF7L2, IGFBP5 |
| KEGG_PATHWAY | rno04060:Cytokine-cytokine receptor interaction | 7 | 0.0319 | IL3, TNFRSF11A, OSMR, IFNA5, TNFRSF8, TNFSF14, CCR1L1 |
| GOTERM_BP_DIRECT | GO:0051607~defense response to virus | 5 | 0.0437 | LOC100911527, ABCC9, IFNA5, BCL2, PMAIP1 |
| GOTERM_BP_DIRECT | GO:0007568~aging | 8 | 0.0453 | CIITA, CDKN1C, SIN3A, BCL2, MADCAM1, IGFBP1, APAF1, IGFBP5 |
| GOTERM_BP_DIRECT | GO:0009408~response to heat | 4 | 0.0469 | CKM, HSP90AA1, BCL2, TRPV3 |
| GOTERM_BP_DIRECT | GO:0061458~reproductive system development | 2 | 0.0701 | PLEKHA5, SPATA22 |
| KEGG_PATHWAY | rno04740:Olfactory transduction | 20 | 0.0772 | OLR1016, OLR1302, OLR159, OLR98, OLR558, OLR1423, OLR703, OLR1413, OLR1024, OLR1630, OLR510, OLR1321, OLR1660, OLR1765, LOC684471, OLR689,OLR1654, OLR921, OLR852, OLR1232 |
| GOTERM_BP_DIRECT | GO:0001503~ossification | 4 | 0.0810 | TNFRSF11A, BCL2, IGF2, SLC26A2 |
| GOTERM_BP_DIRECT | GO:0009266~response to temperature stimulus | 2 | 0.0892 | TOP1, TRPV3 |

Supplementary table 3

| **Hippocampus** | | | | |
| --- | --- | --- | --- | --- |
| **Category** | **Term** | **Count** | **PValue** | **Genes** |
| UP_SEQ_FEATURE | disulfide bond | 58 | 2.00E-09 | OPRM1, TAAR7D, MMP8, CRP, TNFSF15, SPINK1, OLR1873, BDNF, AGTR2, DPEP3, TAAR1, TAAR2, KLRB1, SCN2B, ZP2, TAAR8A, F9, AMY2A3, OLR1496, AADAC, C8B, IGSF5, SSTR2, PRL3D1, PRL4A1, NPTN, ADAM18, DEFB43, LOC312273, KIR3DL1, GC, ABCA7, OLR1078, RUP2, CYSLTR2, GALNT5, PRL3C1, CCL5, PCSK1, MUSK, PIP, PRL7A3, MUP5, KLK7, PNLIPRP1, HAVCR1, PSBPC2, PRL3B1, GABRA6, RGD1563136, A1BG, P2RY13, SLC18A2, IGFBP1, AREG, KLRB1C, HABP2, IGFBP5 |
| GOTERM_MF_DIRECT | GO:0033038~bitter taste receptor activity | 6 | 0.00256 | TAS2R107, TAS2R106, TAS2R105, TAS2R114, TAS2R102, TAS2R130 |
| UP_SEQ_FEATURE | topological domain:Extracellular | 38 | 0.00325 | OPRM1, OLR1078, ABCA7, CYSLTR2, TAAR7D, TNFSF15, TAS2R114, SLC26A2, OLR1873, AGTR2, MUSK, ENTPD8, TAAR1, TAAR2, KLRB1, HAVCR1, SCN2B, ZP2, GABRA6, TAAR8A, TAS2R102, OLR1496, HCST, IGSF5, P2RY13, SSTR2, SLC26A5, TAS2R107, GRM8, LAX1, NPHS2, TAS2R105, CD99L2, NPTN, ADAM18, KLRB1C, KIR3DL1, TAS2R130 |
| GOTERM_MF_DIRECT | GO:0030594~neurotransmitter receptor activity | 6 | 0.00328 | OLR1632, OLR1063, OLR517, OLR1085, OLR1081, OLR1095 |
| GOTERM_MF_DIRECT | GO:0004993~G-protein coupled serotonin receptor activity | 6 | 0.00369 | OLR1632, OLR1063, OLR517, OLR1085, OLR1081, OLR1095 |
| INTERPRO | IPR000198:Rho GTPase-activating protein domain | 8 | 0.00400 | DLC1, ARHGAP31, LOC678817, CHN2, LOC100365525, DEPDC1, ARHGAP24, ARAP2 |
| UP_KEYWORDS | Secreted | 44 | 0.00422 | CTS7, GC, ODAM, SPINK13, SVS4, RUP2, GRPCA, CRP, MMP8, PRL3C1, SPINK1, PRL7D1, CCL5, OTOS, ADCYAP1, BDNF, APOF, PIP, PRL7A3, COL8A2, RNASE13, PRL8A2, MUP5, MUC2, PNLIPRP1, PRL7A4, ZP2, PSBPC2, BPIFA1, PRL3B1, IL7, F9, AMY2A3, A1BG, SCGB2A1, C8B, PRL3D1, PRL4A1, DEFB43, IGFBP1, LOC312273, CSN1S2A, IGFBP5, HABP2 |
| UP_KEYWORDS | Hormone | 9 | 0.00540 | PRL7A4, PRL3D1, PRL3B1, PRL4A1, PRL3C1, PRL7A3, PRL7D1, PRL8A2, ADCYAP1 |
| COG_ONTOLOGY | Secondary metabolites biosynthesis, transport, and catabolism | 6 | 0.00576 | CYP24A1, CYP2J4, CYP1A1, CYP7A1, CYP4A8, CYP2A3 |
| SMART | SM00324:RhoGAP | 7 | 0.00591 | DLC1, ARHGAP31, LOC678817, CHN2, LOC100365525, ARHGAP24, ARAP2 |
| INTERPRO | IPR004072:Vomeronasal receptor, type 1 | 9 | 0.00782 | VOM1R83, VOM1R84, VOM1R89, VOM1R106, VOM1R35, VOM1R14, VOM1R34, VOM1R81, VOM1R5 |
| GOTERM_MF_DIRECT | GO:0001594~trace-amine receptor activity | 4 | 0.00892 | TAAR7D, TAAR8A, TAAR1, TAAR2 |
| GOTERM_MF_DIRECT | GO:0008527~taste receptor activity | 4 | 0.00892 | TAS2R107, TAS2R105, TAS2R114, TAS2R130 |
| GOTERM_MF_DIRECT | GO:0005179~hormone activity | 9 | 0.00900 | PRL7A4, PRL3D1, PRL3B1, PRL4A1, PRL3C1, PRL7A3, PRL7D1, PRL8A2, ADCYAP1 |
| INTERPRO | IPR009132:Trace amine receptor | 4 | 0.00991 | TAAR7D, TAAR8A, TAAR1, TAAR2 |
| INTERPRO | IPR000010:Proteinase inhibitor I25, cystatin | 5 | 0.0102 | CSTL1, STFA3L1, LOC689230, STFA3, CST9L |
| GOTERM_CC_DIRECT | GO:0005576~extracellular region | 31 | 0.0119 | ODAM, RUP2, GRPCA, CRP, PRL3C1, PRL7D1, OTOS, ADCYAP1, BDNF, GLIPR1, PIP, PRL7A3, RNASE13, PRL8A2, RS1, MUP5, PNLIPRP1, PRL7A4, ZP2, PSBPC2, BPIFA1, PRL3B1, A1BG, C8B, SCGB2A1, PRL3D1, PRL4A1, DEFB43, CSN1S2A, HABP2, IGFBP5 |
| GOTERM_BP_DIRECT | GO:0033574~response to testosterone | 7 | 0.0126 | LOC100912608, HAVCR1, HOXA11, S100VP, CRP, CYP4A8, HOXD13 |
| GOTERM_MF_DIRECT | GO:0016503~pheromone receptor activity | 9 | 0.0129 | VOM1R83, VOM1R84, VOM1R89, VOM1R106, VOM1R35, VOM1R14, VOM1R34, VOM1R81, VOM1R5 |
| GOTERM_BP_DIRECT | GO:0097062~dendritic spine maintenance | 3 | 0.0138 | MTMR2, TANC1, ITGA3 |
| GOTERM_BP_DIRECT | GO:0007608~sensory perception of smell | 15 | 0.0141 | OLR850, OLR392, NXNL2, OLR234, OLR555, OLR833, OLR1393, OLR927, GRM8, OLR809, LOC684471, OLR865, OLR847, OLR853, OLR205 |
| INTERPRO | IPR008936:Rho GTPase activation protein | 8 | 0.0165 | DLC1, ARHGAP31, LOC678817, CHN2, LOC100365525, DEPDC1, ARHGAP24, ARAP2 |
| GOTERM_BP_DIRECT | GO:0070233~negative regulation of T cell apoptotic process | 3 | 0.0228 | PIP, CCL5, CD27 |
| INTERPRO | IPR001713:Proteinase inhibitor I25A, stefin A | 3 | 0.0229 | STFA3L1, LOC689230, STFA3 |
| INTERPRO | IPR000863:Sulfotransferase domain | 5 | 0.0238 | LOC100910057, HS3ST3A1, SULT1A1, CHST4, RGD1559960 |
| UP_KEYWORDS | Serine protease | 10 | 0.0243 | KLK7, PCSK1, KLK9, OVCH2, PRSS44, F9, PCSK6, KLK15, LOC312273, HABP2 |
| SMART | SM00043:CY | 4 | 0.0268 | CSTL1, STFA3L1, LOC689230, STFA3 |
| SMART | SM00020:Tryp_SPc | 9 | 0.0291 | KLK7, KLK9, OVCH2, PRSS44, F9, KLK15, LOC312273, KLK5L, HABP2 |
| **Hypothalamus** | |  |  |  |
| UP_KEYWORDS | Transport | 45 | 0.00303 | SLC44A1, KCNC3, TXN2, SLC20A2, KCNAB1, NUP62CL, RAB1B, KCNIP4, ACBD5, APOA2, SLC2A6, KCNS1, SLC48A1, OSBPL1A, GRID2, EXOC4, RAB11B, SLC25A1, SLC25A45, RAB6A, NALCN, EHD3, SLC4A5, HCN2, SLC12A9, SLC12A5, CACNG6, SLC22A23, ATP5F1, MICALL1, LIN7A, SENP2, RGD1559629, CLIC4, TOM1L2, LASP1, ARCN1, ATP5C1, SORT1, SLC25A19, OSBPL11, SCN8A, CLCN6, ASNA1, CACNA1A |
| KEGG_PATHWAY | rno04724:Glutamatergic synapse | 9 | 0.00322 | PLA2G4A, ADCY1, GLUL, GRM3, PLCB3, PPP3R1, PLCB1, PLA2G4B, CACNA1A |
| KEGG_PATHWAY | rno04310:Wnt signalling pathway | 10 | 0.00324 | SFRP5, SENP2, PLCB3, CSNK1E, JUN, PPP3R1, MAPK9, PLCB1, AXIN2, TBL1X |
| KEGG_PATHWAY | rno04912:GnRH signalling pathway | 8 | 0.00351 | PLA2G4A, ADCY1, PLCB3, GRB2, JUN, MAPK9, PLCB1, PLA2G4B |
| GOTERM_CC_DIRECT | GO:0016020~membrane | 68 | 0.00366 | SEC1, ABCF3, SLC44A1, KCNAB1, PCDHGA5, FOXO3, MCF2L, ACBD5, KCNIP4, RNF141, PICALM, MGRN1, PAK2, ANK3, PIGG, GRID2, ABHD13, RAB6A, ATP6V0D1, PLCB1, RAP2C, CMAS, CDK9, GZMB, PKD2L1, TMEM131, FAM120A, CEP350, MED16, BACE1, CD81, ARCN1, CFL1, VEGFA, ACAP2, HSD11B1, ATP5C1, MGEA5, ERC2, ADD3, GRB2, STK11, ENPP4, CTNND1, LRIG2, G6PC3, CALU, STAU1, SEMA5A, PEF1, BCAP29, GYS1, EXOC4, KDM3A, SLC31A2, HCN2, LOC102546892, ACY3, KL, CNKSR3, ATP5F1, DPYSL2, LIN7A, MPHOSPH9, PEX11B, SYNM, SCN8A, NCOR1 |
| GOTERM_CC_DIRECT | GO:0000118~histone deacetylase complex | 5 | 0.00456 | TBL1X, NCOR1, RERE, NRIP1, ELMSAN1 |
| GOTERM_BP_DIRECT | GO:0071805~potassium ion transmembrane transport | 9 | 0.00541 | HCN2, KCNS1, SLC12A9, KCNC3, KCNAB1, SLC12A5, NALCN, PKD2L1, KCNIP4 |
| KEGG_PATHWAY | rno04152:AMPK signalling pathway | 9 | 0.00587 | G6PC, STK11, GYS1, ADIPOR2, RAB11B, PIK3CA, CREB5, FOXO3, G6PC3 |
| GOTERM_BP_DIRECT | GO:0009395~phospholipid catabolic process | 4 | 0.00680 | PLA2G4A, APOA2, PLCB3, PLA2G4B |
| UP_KEYWORDS | Ion transport | 21 | 0.00739 | HCN2, KCNC3, KCNAB1, SLC20A2, SLC22A23, CACNG6, SLC12A5, NUP62CL, ATP5F1, KCNIP4, KCNS1, RGD1559629, LASP1, CLIC4, GRID2, ATP5C1, SCN8A, NALCN, CLCN6, CACNA1A, SLC4A5 |
| KEGG_PATHWAY | rno04921:Oxytocin signalling pathway | 10 | 0.00739 | PLA2G4A, ADCY1, PLCB3, JUN, CACNG6, PPP3R1, PPP1R12A, PIK3CA, PLCB1, PLA2G4B |

Supplementary table 4

| **Nr.** | **Gene** | **Name** | **Description/Comments/Relevance of the gene^^[[2]](#footnote-2)^^** |
| --- | --- | --- | --- |
|  | Olr379  Olr522  Olr655  Olr841 | Olfactory receptors | These genes are involved in stress response and perception of smell.  OR gene family already sufficiently covered. No renewed qPCR verification. Will be excluded from the current gene selection. The previous qPCR verification of these genes is included in the overall interpretation of the genes to be verified below. |
| Target gene selection based on table “GSEA compensation analyses“ (FC > 1.5, p < 0.2; Filter: compensation)  Query: Which OVX-induced change is being countered by iCR? | | | |
| 1 | AVPR1A  (Hi) | arginine vasopressin receptor 1A | The protein encoded by this gene acts as receptor for arginine vasopressin. This receptor belongs to the subfamily of G-protein coupled receptors which includes AVPR1B, V2R and OXT receptors. Its activity is mediated by G proteins which stimulate a phosphatidylinositol-calcium second messenger system. The receptor mediates cell contraction and proliferation, platelet aggregation, release of coagulation factor and glycogenolysis. |
| 2 | GAL  (Hi) | galanin and GMAP prepropeptide | This gene encodes a neuroendocrine peptide that is widely expressed in the central and peripheral nervous systems and also the gastrointestinal tract, pancreas, adrenal gland and urogenital tract. The encoded protein is a precursor that is proteolytically processed to generate two mature peptides: galanin and galanin message-associated peptide (GMAP). Galanin has diverse physiological functions including nociception, feeding and energy homeostasis, osmotic regulation and water balance. GMAP has been demonstrated to possess antifungal activity and hypothesized to be part of the innate immune system |
| 3 | CALCA  (Hi) | calcitonin related polypeptide alpha | This gene encodes the peptide hormones calcitonin, calcitonin gene-related peptide and katacalcin by tissue-specific alternative RNA splicing of the gene transcripts and cleavage of inactive precursor proteins. Calcitonin is involved in calcium regulation and acts to regulate phosphorus metabolism. Calcitonin gene-related peptide functions as a vasodilator and as an antimicrobial peptide while katacalcin is a calcium-lowering peptide. Multiple transcript variants encoding different isoforms have been found for this gene. |
| 4 | HCRT  (Hi) | hypocretin neuropeptide precursor | This gene encodes a hypothalamic neuropeptide precursor protein that gives rise to two mature neuropeptides, orexin A and orexin B, by proteolytic processing. Orexin A and orexin B, which bind to orphan G-protein coupled receptors HCRTR1 and HCRTR2, function in the regulation of sleep and arousal. This neuropeptide arrangement may also play a role in feeding behavior, metabolism, and homeostasis. |
| 5 | PNOC  (Hi) | prepronociceptin | This gene encodes a preproprotein that is proteolytically processed to generate multiple protein products. These products include nociceptin, nocistatin, and orphanin FQ2 (OFQ2). Nociceptin, also known as orphanin FQ, is a 17-amino acid neuropeptide that binds to the nociceptin receptor to induce increased pain sensitivity, and may additionally regulate body temperature, learning and memory, and hunger. Another product of the encoded preproprotein, nocistatin, may inhibit the effects of nociceptin. |
| 6 | IL5  (Hi) | Interleukin 5 | This gene encodes a cytokine that acts as a growth and differentiation factor for both B cells and eosinophils. The encoded cytokine plays a major role in the regulation of eosinophil formation, maturation, recruitment and survival. The increased production of this cytokine may be related to pathogenesis of eosinophil-dependent inflammatory diseases. This cytokine functions by binding to its receptor, which is a heterodimer, whose beta subunit is shared with the receptors for interleukin 3 (IL3) and colony stimulating factor 2 (CSF2/GM-CSF). This gene is located on chromosome 5 within a cytokine gene cluster which includes interleukin 4 (IL4), interleukin 13 (IL13), and CSF2 . This gene, IL4, and IL13 may be regulated coordinately by long-range regulatory elements spread over 120 kilobases on chromosome 5q31. |
| 7 | BCL2  (Hy) | BCL2, apoptosis regulator | This gene encodes an integral outer mitochondrial membrane protein that blocks the apoptotic death of some cells such as lymphocytes. Constitutive expression of BCL2, such as in the case of translocation of BCL2 to Ig heavy chain locus, is thought to be the cause of follicular lymphoma. Alternative splicing results in multiple transcript variants. |
| 8 | IGFBP1  (Hy) | insulin like growth factor binding protein 1 | This gene is a member of the insulin-like growth factor binding protein (IGFBP) family and encodes a protein with an IGFBP N-terminal domain and a thyroglobulin type-I domain. The encoded protein, mainly expressed in the liver, circulates in the plasma and binds both insulin-like growth factors (IGFs) I and II, prolonging their half-lives and altering their interaction with cell surface receptors. This protein is important in cell migration and metabolism. Low levels of this protein may be associated with impaired glucose tolerance, vascular disease and hypertension in human patients. |
| 9 | IGFBP5  (Hy) | insulin like growth factor binding protein 5 |  |
| 10 | IL3  (Hy) | Interleukin 3 | The protein encoded by this gene is a potent growth promoting cytokine. This cytokine is capable of supporting the proliferation of a broad range of hematopoietic cell types. It is involved in a variety of cell activities such as cell growth, differentiation and apoptosis. This cytokine has been shown to also possess neurotrophic activity, and it may be associated with neurologic disorders |
| 11 | TRPV3  (Hy) | transient receptor potential cation channel subfamily V member 3 | This gene product belongs to a family of nonselective cation channels that function in a variety of processes, including temperature sensation and vasoregulation. The thermosensitive members of this family are expressed in subsets of sensory neurons that terminate in the skin, and are activated at distinct physiological temperatures. This channel is activated at temperatures between 22 and 40 degrees C. This gene lies in close proximity to another family member gene on chromosome 17, and the two encoded proteins are thought to associate with each other to form heteromeric channels. Multiple transcript variants encoding different isoforms have been found for this gene. |
| Target gene selection based on ”GSEA iCR exclusive” (FC > 1.5, p < 0.2);  Query: What is changed exclusively by iCR? | | | |
| 12 | PRL7A3  (Hi) | prolactin family 7, subfamily a, member 3 | involved in the control of maternal and fetal adaptations to pregnancy |
| 13 | PLCB1  (Hy) | phospholipase C beta 1 | The protein encoded by this gene catalyzes the formation of inositol 1,4,5-trisphosphate and diacylglycerol from phosphatidylinositol 4,5-bisphosphate. This reaction uses calcium as a cofactor and plays an important role in the intracellular transduction of many extracellular signals |
| 14 | MAPK9  (Hy) | mitogen-activated protein kinase 9 | The protein encoded by this gene is a member of the MAP kinase family. MAP kinases act as an integration point for multiple biochemical signals, and are involved in a wide variety of cellular processes such as proliferation, differentiation, transcription regulation and development. This kinase targets specific transcription factors, and thus mediates immediate-early gene expression in response to various cell stimuli. It is most closely related to MAPK8, both of which are involved in UV radiation induced apoptosis, thought to be related to the cytochrome c-mediated cell death pathway. This gene and MAPK8 are also known as c-Jun N-terminal kinases. This kinase blocks the ubiquitination of tumor suppressor p53, and thus it increases the stability of p53 in nonstressed cells. Studies of this gene's mouse counterpart suggest a key role in T-cell differentiation. |
| 15 | ADCY1  (Hy) | adenylate cyclase 1 | This gene encodes a member of the adenylate cyclase gene family that is primarily expressed in the brain. This protein is regulated by calcium/calmodulin concentration and may be involved in brain development. |
| 16 | G6PC  (Hy) | glucose-6-phosphatase catalytic subunit | Glucose-6-phosphatase (G6Pase) is a multi-subunit integral membrane protein of the endoplasmic reticulum that is composed of a catalytic subunit and transporters for G6P, inorganic phosphate, and glucose. This gene (G6PC) is one of the three glucose-6-phosphatase catalytic-subunit-encoding genes in human: G6PC, G6PC2 and G6PC3. Glucose-6-phosphatase catalyzes the hydrolysis of D-glucose 6-phosphate to D-glucose and orthophosphate and is a key enzyme in glucose homeostasis, functioning in gluconeogenesis and glycogenolysis. |
| 17 | CACNA1A (Hy) | calcium voltage-gated channel subunit alpha1 A | Voltage-dependent calcium channels mediate the entry of calcium ions into excitable cells, and are also involved in a variety of calcium-dependent processes, including muscle contraction, hormone or neurotransmitter release, and gene expression |
| Target genes, which are not included in the above mentioned lists, but are generally important  Query: General importance in regard to recent scientific work [[10-19](#_ENREF_10)] | | | |
| 18 | ESR1 | estrogen receptor 1 (alpha) | This gene encodes an estrogen receptor, a ligand-activated transcription factor composed of several domains important for hormone binding, DNA binding, and activation of transcription. The protein localizes to the nucleus where it may form a homodimer or a heterodimer with estrogen receptor 2. Estrogen and its receptors are essential for sexual development and reproductive function, but also play a role in other tissues such as bone. Estrogen receptors are also involved in pathological processes including breast cancer, endometrial cancer, and osteoporosis. Alternative promoter usage and alternative splicing result in dozens of transcript variants, but the full-length nature of many of these variants has not been determined |
| 19 | ESR2 | estrogen receptor 2 (beta) | This gene encodes a member of the family of estrogen receptors and superfamily of nuclear receptor transcription factors. The gene product contains an N-terminal DNA binding domain and C-terminal ligand binding domain and is localized to the nucleus, cytoplasm, and mitochondria. Upon binding to 17beta-estradiol or related ligands, the encoded protein forms homo- or hetero-dimers that interact with specific DNA sequences to activate transcription. Some isoforms dominantly inhibit the activity of other estrogen receptor family members. Several alternatively spliced transcript variants of this gene have been described, but the full-length nature of some of these variants has not been fully characterized. |
| 20 | OPRM1 | opioid receptor mu 1 | This gene encodes one of at least three opioid receptors in humans; the mu opioid receptor (MOR). The MOR is the principal target of endogenous opioid peptides and opioid analgesic agents such as beta-endorphin and enkephalins. The MOR also has an important role in dependence to other drugs of abuse, such as nicotine, cocaine, and alcohol via its modulation of the dopamine system. The NM_001008503.2:c.118A>G allele has been associated with opioid and alcohol addiction and variations in pain sensitivity but evidence for it having a causal role is conflicting. Multiple transcript variants encoding different isoforms have been found for this gene. Though the canonical MOR belongs to the superfamily of 7-transmembrane-spanning G-protein-coupled receptors some isoforms of this gene have only 6 transmembrane domains. |
| 21 | KISS1 | KiSS-1 metastasis-suppressor | This gene is a metastasis suppressor gene that suppresses metastases of melanomas and breast carcinomas without affecting tumorigenicity. The encoded protein may inhibit chemotaxis and invasion and thereby attenuate metastasis in malignant melanomas. Studies suggest a putative role in the regulation of events downstream of cell-matrix adhesion, perhaps involving cytoskeletal reorganization. A protein product of this gene, kisspeptin, stimulates gonadotropin-releasing hormone (GnRH)-induced gonadotropin secretion and regulates the pubertal activation of GnRH neurons. A polymorphism in the terminal exon of this mRNA results in two protein isoforms. An adenosine present at the polymorphic site represents the third position in a stop codon. When the adenosine is absent, a downstream stop codon is utilized and the encoded protein extends for an additional seven amino acid residues. |
| 22 | TAC3 | Tachykinin 3 | This gene encodes a member of the tachykinin family of secreted neuropeptides. The encoded preproprotein is proteolytically processed to generate the mature peptide, which is primarily expressed in the central and peripheral nervous systems and functions as a neurotransmitter. This peptide is the ligand for the neurokinin-3 receptor. This protein is also expressed in the outer syncytiotrophoblast of the placenta and may be associated with pregnancy-induced hypertension and pre-eclampsia. Mutations in this gene are associated with normosmic hypogonadotropic hypogonadism. Alternative splicing results in multiple transcript variants, at least one of which encodes an isoform that is proteolytically processed. |

1. When members of two independent groups can fall into one of two mutually exclusive categories, Fisher Exact test is used to determine whether the proportions of those falling into each category differs by group. In DAVID annotation system, Fisher Exact is adopted to measure the gene-enrichment in annotation terms.

   A Hypothetical Example:

   In human genome background (30,000 gene total), 40 genes are involved in p53 signaling pathway. A given gene list has found that 3 out of 300 belong to p53 signaling pathway. Then we ask the question if 3/300 is more than random chance comparing to the human background of 40/30000.

   Fisher Exact P-Value = 0.008 (using 3 instead of 3-1). Since P-Value <= 0.01, this user gene list is specifically associated (enriched) in p53 signaling pathway than random chance.

   However, EASE Score is more conservative to examine the situation. EASE Score = 0.06 (using 3-1 instead of 3). Since P-Value > 0.01, this user gene list is specifically associated (enriched) in p53 signaling pathway no more than random chance. [↑](#footnote-ref-1)
2. Information taken from the NCBI “gene” database (<https://www.ncbi.nlm.nih.gov/gene/>) [↑](#footnote-ref-2)
